# Increased Adenovirus Type 5 Mediated Transgene Expression Due to RhoB Down-Regulation

**DOI:** 10.1371/journal.pone.0086698

**Published:** 2014-01-22

**Authors:** Dragomira Majhen, Nikolina Stojanović, Dunja Vukić, Chantal Pichon, Chloé Leduc, Maja Osmak, Andreja Ambriović-Ristov

**Affiliations:** 1 Division of Molecular Biology, Ruđer Bošković Institute, Zagreb, Croatia; 2 Centre de Biophysique Moléculaire CNRS-UPR4301 Affiliated to the Université d'Orléans, Orléans, France; Centro Nacional de Biotecnologia (CNB-CSIC), Spain

## Abstract

Adenovirus type 5 (Ad5) is a non-enveloped DNA virus frequently used as a gene transfer vector. Efficient Ad5 cell entry depends on the availability of its primary receptor, coxsackie and adenovirus receptor, which is responsible for attachment, and integrins, secondary receptors responsible for adenovirus internalization via clathrin-mediated endocytosis. However, efficacious adenovirus-mediated transgene expression also depends on successful trafficking of Ad5 particles to the nucleus of the target cell. It has been shown that changes occurring in tumor cells during development of resistance to anticancer drugs can be beneficial for adenovirus mediated transgene expression. In this study, using an in vitro model consisting of a parental cell line, human laryngeal carcinoma HEp2 cells, and a cisplatin-resistant clone CK2, we investigated the cause of increased Ad5-mediated transgene expression in CK2 as compared to HEp2 cells. We show that the primary cause of increased Ad5-mediated transgene expression in CK2 cells is not modulation of receptors on the cell surface or change in Ad5wt attachment and/or internalization, but is rather the consequence of decreased RhoB expression. We propose that RhoB plays an important role in Ad5 post-internalization events and more particularly in Ad5 intracellular trafficking. To the best of our knowledge, this is the first study showing changed Ad5 trafficking pattern between cells expressing different amount of RhoB, indicating the role of RhoB in Ad5 intracellular trafficking.

## Introduction

Adenovirus-based vectors are leading vectors used in gene therapy clinical trials today. Human adenovirus type 5 (Ad5) is a dsDNA virus with an icosahedral, non-enveloped capsid composed of 240 hexon protein trimers and 12 pentons, each of which comprising a pentameric penton base and a trimeric fiber protein that protrudes from the apex of the penton base [1]. Ad5 infection begins with high-affinity binding of the fiber protein to the coxsackie-adenovirus receptor (CAR) on the cell surface [2]. Interaction between RGD motifs of the penton base and cell-surface integrins (αvβ3, αvβ5, αvβ1, α5β1 and α3β1) then triggers internalization of the viral particle [3], [4], [5], [6].

In order to enter the host cell, adenoviruses use existing cell entry pathways. Ad5 internalization is mostly mediated by dynamin- and clathrin-dependent receptor-mediated endocytosis [7], although there is evidence that some capsid-modified Ad5-based vectors can enter the cell by using lipid raft- and caveolae-mediated endocytosis [8]. After being internalized, Ad5 continues part of its intracellular journey in the endosome. It is widely accepted that escape of Ad5 from the endosome is induced by endosomal acidification. Lowering pH in the endosome allows dismantling of the Ad5 capsid and release of the membrane-lytic internal protein VI, which then triggers penetration of the endosome. It has also been shown that integrin αvβ5 plays an important role in the release of Ad5 from the endosome [9], [10], [11]. Once in the cytoplasm, adenovirus encounters complex networks of organelles and proteins, which severely impair diffusive mobility. Therefore, intracellular trafficking of Ad5 cannot rely on simple diffusion, but rather involves active transport. After being liberated from the endosome, adenovirus binds directly to the microtubule minus end-directed motor dynein and is transported all the way to the nucleus [12]. The process of adenovirus endocytosis is regulated by lipid kinases and actin-modulating small GTPases, and has been shown to require assembly of the actin cytoskeleton, an event initiated by activation of PI3K and, subsequently, Rac and Cdc42, members of the Rho GTPase family [13].

Rho GTPases are members of the Ras superfamily of monomeric GTP-binding proteins that have an important role in regulating the actin cytoskeleton and membrane trafficking. While RhoA, Rac and Cdc42 localize to the plasma membrane and are involved in receptor internalization, RhoB is found both at the plasma membrane and endosomes and has been suggested to regulate endosomal traffic [14]. RhoB is involved in traffic to the cell surface, nucleus, or lysosome, and/or activation of a number of signaling molecules, such as RTKs, Akt and Src [15], [16], [17]. It has recently been reported that activated RhoB promotes the polymerization of an actin coat around endosomes and association of these vesicles to subcortical actin cables, thus effectively inhibiting further endosomal transport [18]. It has been also proposed that RhoB plays a key role in the recycling/degradation sorting decision in CXCR2 receptor trafficking [19]. RhoB was found to play a role in entry of Ebola virus and vesicular stomatitis virus, two enveloped viruses that use different receptors but converge on a common Rho-mediated pathway, suggesting that this may be a route of entry utilized by many different viruses [20]. From the literature it is known that interfering with actin can impede adenovirus-mediated transduction [21], [22]; at present, however, no data are available concerning the role of RhoB in adenovirus entry.

Second only to heart disease, cancer is a major medical cause of death in the human population. Standard cancer treatment includes surgery, chemotherapy, radiation therapy, and immunotherapy. A very important drawback of chemotherapy, which is often used as a treatment of choice, is the development of chemoresistance. Cancer cells develop resistance to the applied anti-cancer drug through various mechanisms, which in many cases allow them to escape the toxic effect of various anti-cancer drugs, in spite of their different chemical structures and modes of action. Gene therapy, though originally designed as a treatment for monogenetic illness, has emerged as an alternative strategy in cancer treatment. Adenovirus-based vectors are particularly promising, as they can be successfully combined with other therapies. In the literature a growing number of studies report encouraging results obtained by combined use of adenoviruses, whether replication deficient or conditionally replicating, with chemotherapy [23], [24], [25]. An attractive feature of the combined approach is that some drug treatments enhance adenovirus infection of cancer cells by changing expression of receptors involved in adenovirus entry, such as CAR [26]. Furthermore, in cell clones resistant to multiple anti-cancer drugs, certain changes that increase Ad5-mediated transgene expression have been characterized, including up-regulation of CAR and integrin αvβ3 in a human laryngeal carcinoma cells resistant to cisplatin CA3_ST_ [27] or increased activity of a Rous sarcoma virus promoter driving Ad5-mediated transgene expression in human laryngeal carcinoma cells resistant to vincristine, [28].

In our laboratory we have developed a cisplatin-resistant cell line, CK2, by chronic exposure of a human laryngeal carcinoma cell line, HEp2, to cisplatin [29]. CK2 cells differ from the parental cell line both in morphology and in the formation of cell–cell adherens junctions in the resistant clone [30]. Moreover, in CK2 cells the down-regulation of RhoB has recently been reported to be one of the causes of drug resistance [31]. We have observed that Ad5-mediated transgene expression is higher in the cisplatin-resistant CK2 cell line than in parental HEp2 cells. In the present study we have investigated the molecular mechanisms responsible for this effect. We show here that the observed difference in Ad5-mediated transgene expression is not simply the consequence of differential expression of receptors involved in adenovirus entry, but is principally due to altered constitutive RhoB expression. We show that silencing RhoB expression, in both parental HEp2 and cisplatin-resistant CK2 cell lines, dramatically increases Ad5-mediated transgene expression regardless of receptor usage, indicating that RhoB might have a general role in the post-binding steps of Ad5 infection and, more particlularly, in intracellular trafficking.

## Materials and Methods

### Cell Lines

Human laryngeal carcinoma (HEp2) cells were obtained from a cell culture bank (GIBCO/BRL Invitrogen, Germany) and grown in Dulbecco’s modified Eagle’s medium (DMEM) supplemented with 10% bovine serum (Invitrogen, Germany) at 37°C with 5% CO_2_ in a humidified atmosphere. The development of HEp2-derived CK2 cells that are resistant to cisplatin (referred to in the text as cisplatin-resistant cells) has been previously described [29], [32]. Before using the CK2 cells in this study, the cells were tested for cisplatin resistance. As reported previously [32], CK2 cells were approximately 1.5- to 2-fold more resistant to cisplatin than HEp2.

### Adenovirus Vectors

All replication-deficient adenovirus vectors used in this study are based on adenovirus type 5 and have been previously described as listed in Table 1. All vectors were amplified in human embryonic kidney (HEK-293) cells, banded in CsCl gradients and stored at −80°C in aliquots. The virus particle concentration was measured by optical density according to the protocol described by Mittereder et al. [33]. All viruses contain the reporter gene lacZ under the control of the RSV promoter. Adenovirus blue forming units (b.f.u.) were determined in HEK-293 cells as previously described by [34].

**Table 1:** Replication defective Ad5s used in a study.

Adenovirus	Fiber modification		Reference
	HI loop	shaft	
Ad5wt	none	none	[11]
Ad5RGD4C	CDCRGDCFC insertion	none	[11]
Ad5Δ639	none	8 instead of22 repeats	[34]
Ad5Δ639RGD4C	CDCRGDCFC insertion	8 instead of22 repeats	[11]

### Adenovirus Labeling

After purification by banding in CsCl and dialysis against PBS buffer, adenovirus particles were incubated with a 20-fold excess of chemically reactive Alexa488-TFP (Molecular Probes, USA) for 2 hours at room temperature in PBS buffer, pH 7.2. The reaction was stopped by incubation for 5 minutes with a 5-fold excess of lysine over Alexa488-TFP. The labeled vector particles were then purified from excess dye by dialysis using Slide-A-Lyzer 10 K dialysis cassettes (Pierce). The transduction efficiency of the modified vector was analyzed by transduction assay in HEK-293 cells. Alexa488-TFP labeling did not alter transduction efficacy of Ad5wt in these cells.

### Determination of Cell Surface Receptor Expression by Flow Cytometry

Flow cytometry was used to analyze expression of integrin heterodimers αvβ3, αvβ5 and α3β1, as well as integrin subunits αv, α5 and β1, and CAR in HEp2 and CK2 cell lines. Briefly, adherent cells were grown in tissue culture dishes (until 80% confluency), detached with Versene (Invitrogen, USA) and washed twice with PBS. Subsequently cells were incubated on ice with the following antibodies: FITC-conjugated anti-human αvβ3 integrin MAb (23C6, Pharmingen), anti-human αvβ5 integrin MAb (MAB1961, Chemicon), anti-human α3β1 integrin MAb (MAB1992, Chemicon), anti-human β1 integrin MAb (JB1A, Chemicon), anti-human α5 integrin MAb (MAB1956Z, Chemicon), anti-human αv integrin MAb (272-17E6, Merck) and anti-human CAR MAb (RmcB, Upstate Cell Signaling Solutions). The binding of unlabeled primary antibodies was revealed by using PE-conjugated anti-mouse Ig antibody (DAKO, USA) as secondary reagent. Isotype control samples were incubated with FITC-conjugated mouse IgG1, mouse IgG1 (Sigma, Germany) or mouse IgG3 (Invitrogen), depending on the isotype of the MAb requiring the control, followed by PE-conjugated anti-mouse Ig antibody (DAKO, USA) where needed.

### Measurement of Ad5-mediated Transgene Expression

Ad5-mediated transgene expression was measured as described previously [11]. Briefly, 10^4^ cells per well were plated in 96-well plates. Twenty-four hours later, cells were transduced in a final volume of 50 µl (in triplicate) with serial two-fold dilutions of all adenoviruses normalized for physical particle number. After 1 hour, viruses were removed and fresh medium was added. Twenty-four hours after infection, cells were fixed with 0.5% glutaraldehyde and stained for β-gal expression using X-gal as a substrate. The number of stained cells was determined by light microscopy. Cells were counted in each well in which the number of blue cells was between 50 and 150. The results are expressed as the number of β-galactosidase-positive cells normalized to the number of physical particles used for infection.

### Adenovirus Attachment and Internalization Measured by Real Time PCR

Adherent HEp2 and CK2 cells were grown in 35-mm tissue culture dishes (until 80% confluency). Purified Ad5wt, 1000 physical particles per cell (pp/cell), was added to cells and incubated for 40 minutes on ice. To measure attachment, unbound viruses were removed by washing the cells twice with cold trypsin and twice with cold PBS. Cells were then harvested with a cell scraper and pelleted by centrifugation. To measure internalization, unbound viruses were removed and warm growth medium was added. After incubation at 37°C for 40 minutes, cells were washed twice with cold trypsin, trypsinized for 7 minutes at 37°C and, after addition of PBS, immediately pelleted by centrifugation. Total DNA (cellular plus viral) was extracted from cell pellets with commercially available materials (DNeasy Kit, Qiagen) and used to quantify viral DNA.

To measure the extent of viral attachment or internalization, viral DNA was quantified by real-time PCR using a target sequence within the gene encoding hexon and by using the QuantiTect SYBR Green PCR Kit (QIAGEN) and Bio-Rad CFX96 Real Time PCR Detection System (Biorad). The primers used were Hexon-1 (5′- CGCGGTGCGGCTGGTG -3′) and Hexon-2 (5′- TGGCGCATCCCATTCTCC -3′). The DNA content per PCR reaction was normalized by a second real-time PCR assay targeting the GAPDH gene using GAPDH1 (5′-AGA ACA TCA TCC CTG CCT CTA CTG-3′) and GAPDH2 (5′-TGT CGC TGT TGA AGT CAG AGG AGA-3′) primers.

### siRNA Experiments

To silence RhoB, we used Silencer Select Predesigned siRNA (catalog No. 4390817, locus ID 388, Ambion, Austin, TX). As a negative control we used non-targeting control siRNA Silencer Select Predesigned siRNA (Negative control #1, catalog No. 4390844, Ambion). Cells were transfected at a confluency of 30–50% in six-well dishes, using Lipofectamine RNAiMAX reagent (Invitrogen) according to the manufacturer’s protocol for forward transfection. In forward transfections, cells are plated in the wells, and the transfection mix is prepared and added the next day. Twenty-four hours after transfection cells were replated for subsequent experiments, evaluation of Ad5-mediated transgene expression or Western blot. Silencing of RhoB expression was verified by Western blot on whole cell lysates 48 hours after transfection using polyclonal antibody against RhoB (Santa Cruz Biotechnology, Santa Cruz, CA). At the same time cells were infected with Ad5 vectors for measurement of Ad5-mediated transgene expression.

### Confocal Microscopy Studies

HEp2 and CK2 cells (2×10^4^ per coverslip) were seeded in 24-well plates. The next day labeled adenoviruses were added to cells (4×10^4^ pp/cell) and incubated on ice for 60 minutes. For analysis of binding, unbound viruses were removed and cells were fixed. For analysis of internalization, unbound viruses were removed and replaced with warm medium; cells were then incubated at 37°C for 30 minutes prior to fixation. In both cases cells were fixed with 2% paraformaldehyde in PBS for 15 minutes at room temperature. Nuclei were labeled with 5 µM DRAQ5® for 10 minutes at 37°C. Coverslips were slide mounted by using Fluoromount (SouthernBiotech, USA). Confocal laser scanning microscopy (CLSM) analyses were performed using a Zeiss Axiovert 200M microscope coupled with a Zeiss LSM 510 scanning device (Carl Zeiss Co. Ltd., Iena, Germany). Observations were made with a x63 objective.

### Statistical Analysis

Each experiment was repeated at least three times. The data were analyzed by the unpaired Student’s t-test and expressed as means ± standard deviation. Data were considered statistically significant at a p-value of <0.05.

## Results

### Characterization of Receptors Involved in Ad5 Transduction in Parental HEp2 and cisplatin-resistant CK2 Cells

**Figure 1:**
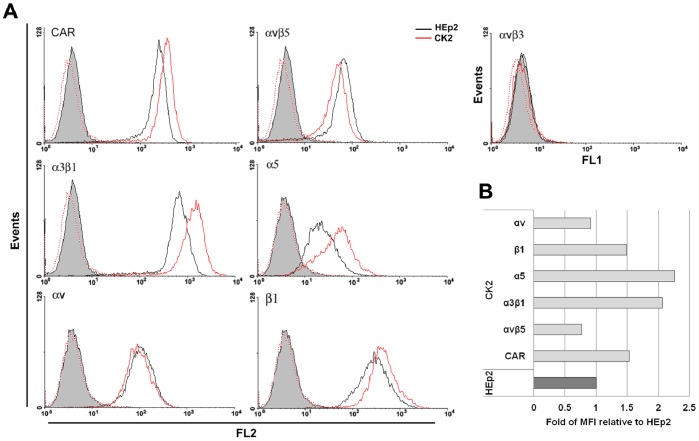
Cell surface levels of CAR, integrin heterodimers αvβ3, αvβ5 and α3β1, integrin subunits α5, αv and β1 on HEp2 and CK2 cells. Cells were detached by Versene and incubated with murine monoclonal antibodies or isotype-matched antibody as a negative control. After incubation with the secondary reagent (i.e. PE-conjugated anti-mouse antibody), labeled cells were analyzed by flow cytometry. (A). Representative histograms and (B) mean fluorescence intensities relative to HEp2 cells obtained in one of three independent experiments that provided similar results are shown.

Drug-resistant cancer cells may exhibit increased Ad5-mediated transgene expression due to altered expression of receptors involved in Ad5 infection [26], [27]. CK2 cells used in this study were developed in our laboratory by chronic exposure of HEp2 cells to cisplatin [29] and in our hands proved to remain approximately 1.5- to 2-fold more resistant to cisplatin than HEp2, as previously reported [32]. In order to find out whether development of cisplatin resistance in the CK2 cell line was accompanied by changes in the repertoire of the cell surface receptors involved in Ad5 infection, we analyzed their expression in HEp2 and CK2 cells by flow cytometry. Data obtained by flow cytometry are presented as histograms (Figure 1A), with corresponding MFI values expressed as the ratio of the MFI for CK2 and HEp2 cells (Figure 1B). No significant difference in αv integrin subunit expression was found between HEp2 and CK2 cells, although there was a slight increase in expression of CAR and β1 integrin subunit and an approximately 2-fold increase in expression of α3β1 integrin heterodimer and α5 integrin subunit. Like the HEp2 cell line [27], CK2 cells do not express αvβ3 integrin (Figure 1A). The only molecule that was found to be down-regulated in CK2 as compared with HEp2 cells was the integrin heterodimer αvβ5, which is very important not only for Ad5 internalization but also for endosome release and has indeed been shown to be a limiting factor for successful Ad5 internalization [10], [11].

### Ad5-mediated Transgene Expression in Cisplatin-resistant CK2 Cells is Higher than in Parental HEp2 Cell Line

**Figure 2:**
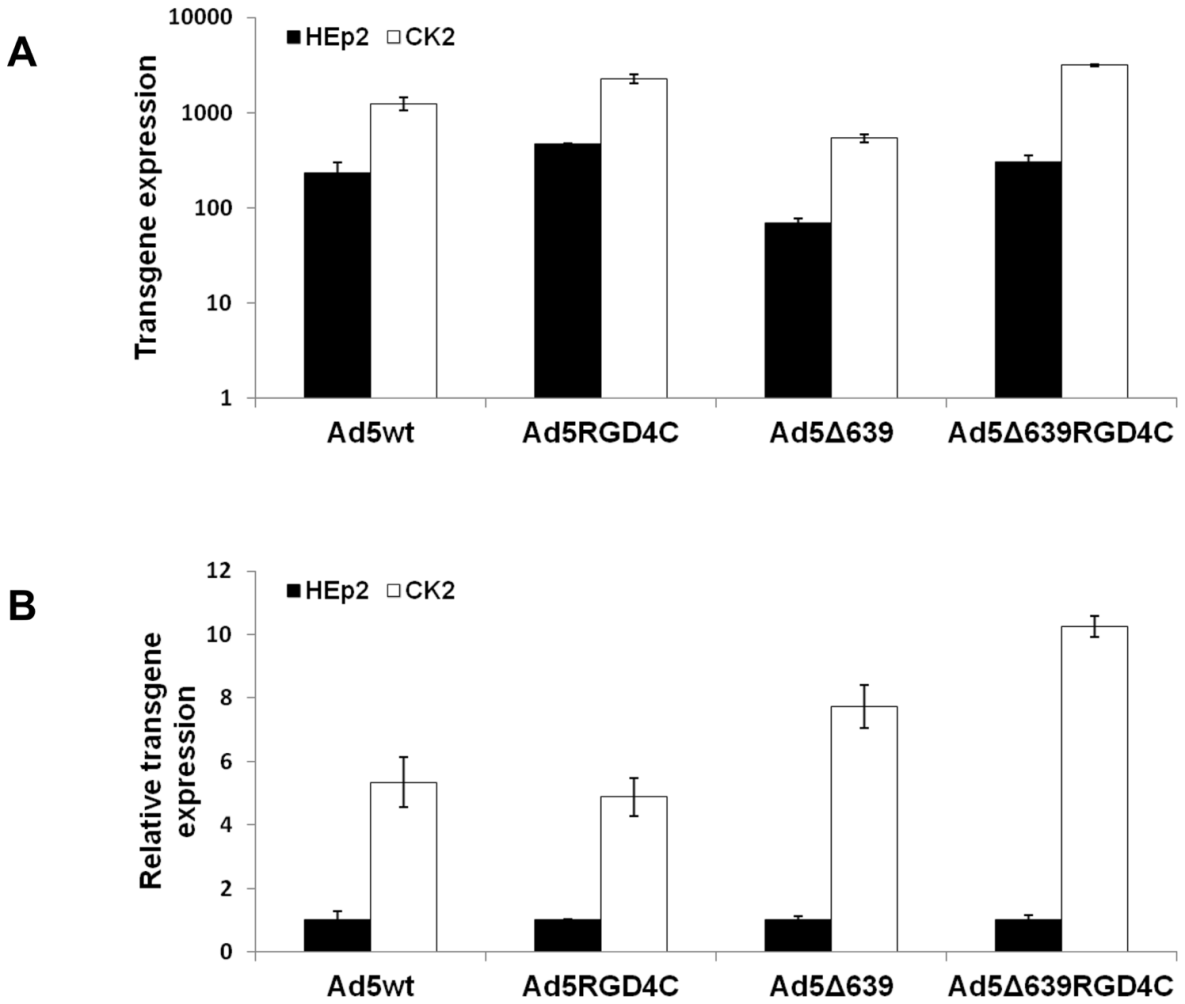
Ad5-mediated transgene expression in cisplatin-resistant CK2 cells is higher than in parental HEp2 cell line. Cells were plated in 96-well plates and, 24 hours later, infected for 1 hour at 37°C with two-fold serial dilutions of Ad5s. Twenty-four hours after infection, cells were stained for β-galactosidase expression. A similar difference was observed over a wide range of dilutions, from 2×10^4^ to 5000 pp/cell; however, only the transgene expression obtained with a MOI of 10^4^ pp/cell is presented. (A). Transgene expression in HEp2 and CK2 cells after transduction with Ad5wt, Ad5RGD4C, Ad5Δ639 and Ad5Δ639RGD4C. (B). Data shown in (A) represented as relative to HEp2 cells. The results presented are representative of three independent experiments with similar results ± standard deviation.

Next, we investigated whether there was a difference in Ad5-mediated transgene expression between cisplatin-resistant CK2 cells and their parental HEp2 cell line. We transduced CK2 cells with four different Ad5 vectors, Ad5wt, Ad5RGD4C, Ad5Δ639 and Ad5Δ639RGD4C, and measured the number of cells expressing the reporter gene product, β-galactosidase. The RGD4C insertion in the HI-loop of fiber protein allows increased adenovirus binding and internalization through αv-integrins [35], which we have confirmed in HEp2 cells [11]. Shortening the Ad5 fiber shaft by introduction of the Δ639 mutation renders transduction by Ad5Δ639 and Ad5Δ639RGD4C vectors independent of CAR [34]. Figure 2A shows that regardless of the modification, all Ad5 vectors tested transduced CK2 cells more efficiently than HEp2 cells, with a fold increase ranging from 5 for Ad5wt and Ad5RGD4C to 8–10 for Ad5Δ639 and Ad5Δ639RGD4C, respectively (Figure 2B). Since the RGD4C-modified viruses Ad5RGD4C and Ad5Δ639RGD4C did not present significant improvement in transgene expression in comparison with their unmodified counterparts, Ad5wt and Ad5Δ639, we can conclude that increased Ad5-mediated transgene expression in CK2 cells is not the consequence of increased expression of RGD-binding integrins αvβ1, αvβ3, αvβ5, and α5β1.

The CK2 cells express 50% more CAR on their cell surface than HEp2 cells. Due to their short and rigid fiber, Ad5Δ639 and Ad5Δ639RGD4C enter cells independently of CAR expression [11], [34]. If the increased Ad5-mediated transgene expression in CK2 cells, as compared with cells, is primarily related to increased CAR expression, we would expect the ratio of transgene expression in CK2 and HEp2 cells to be higher for CAR-dependent (Ad5wt and Ad5RGD4C) than CAR-independent (Ad5Δ639 and Ad5Δ639RGD4C) vectors. We observed, however, that transgene expression was 5.3- and 7.7-fold higher in CK2 cells for Ad5wt and Ad5Δ639, respectively, and 5- and 10-fold higher for Ad5RGD4C and Ad5Δ639RGD4C, respectively. Thus, the magnitude of the increase in transgene expression in CK2 cells was comparable for CAR-dependent and CAR-independent vectors, suggesting that factors other than CAR expression improved Ad5-mediated transgene expression in CK2 cells.

Our group has previously shown that drug-resistance may be accompanied with alteration in promoter activity of the Rous sarcoma virus (RSV) long terminal repeat (LTR), thereby affecting Ad5-mediated transgene expression when transgenes are placed under control of the RSV LTR [28]. To determine whether increased transgene expression might be related to use of the RSV LTR, we measured transgene expression in HEp2 and CK2 cells after transduction with Ad5CMVβgal, which differs from Ad5wt only in that the intermediate/early promoter of CMV drives expression of the lacZ transgene. We observed that CMV-driven transgene expression was increased to a similar extent (5-fold) in CK2 cells (data not shown), and therefore conclude that increased transgene expression in CK2 cells is unlikely to be caused by change in promoter activity.

### Ad5wt Shows Slightly Higher Attachment and Internalization in the CK2 Cell Line

**Figure 3:**
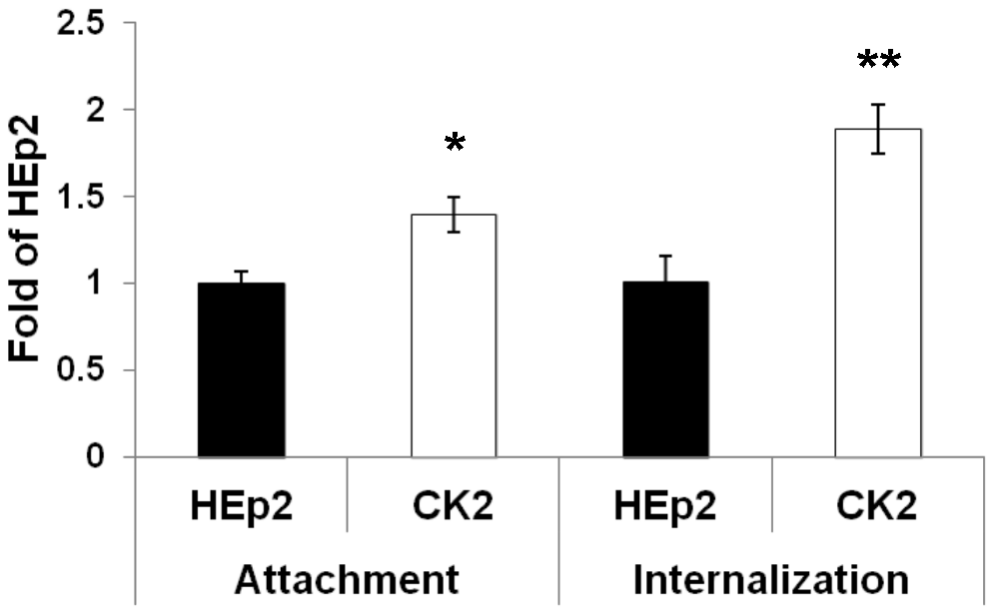
Ad5wt shows slightly higher attachment and internalization in cisplatin-resistant CK2 cells comparing to parental HEp2 cell line. Cells were incubated with Ad5wt at MOI 1000 for 40 minutes on ice. To measure attachment, unbound viruses were removed and cells were scraped off and pelleted. To measure internalization, unbound viruses were removed and cells were incubated at 37°C for 40 minutes, trypsinized and pelleted. Total DNA (cellular plus viral) was extracted from cells and used as a sample for quantification of viral DNA by real-time PCR using a region within the hexon as the target sequence. The cell-derived DNA content per PCR reaction was normalized by a second real-time PCR assay targeting the GAPDH gene. The results presented are representative of three independent experiments with similar results ± standard deviation. Asterisks indicate significant differences (*, P<0.05; **, P<0.01).

As shown in previous sections of this study, certain cell surface molecules involved in adenovirus infection are differentially expressed in the cisplatin-resistant cell line CK2, but the modest differences observed seemed unlikely to be the main cause of increased Ad5-mediated transgene expression in CK2 cells. To determine whether early steps in adenovirus infection were nevertheless altered in the cisplatin-resistant cell line, we compared attachment and internalization of Ad5wt in CK2 and HEp2 cells by real-time PCR. As can be seen in Figure 3, attachment and internalization of Ad5wt were approximately 1.4-fold and 1.8-fold higher, respectively, in CK2 cells than in HEp2 cells (Figure 3). In addition to real-time PCR, we measured binding and internalization by semiquantitative PCR and flow cytometry. Flow cytometry was performed as described previously [28] using a wide range of pp/cell (750–5000). The results were similar to those obtained by real-time PCR (data not shown). While the observed differences in attachment and internalization were statistically significant, we believe that they were too small to account for the large increase in Ad5-mediated transgene expression in the CK2 cell line. This prompted us to investigate other possible mechanisms underlying increased Ad5wt-mediated transgene expression in CK2 cell line.

### Decreasing RhoB Expression Increases Ad5-mediated Transgene Expression

**Figure 4:**
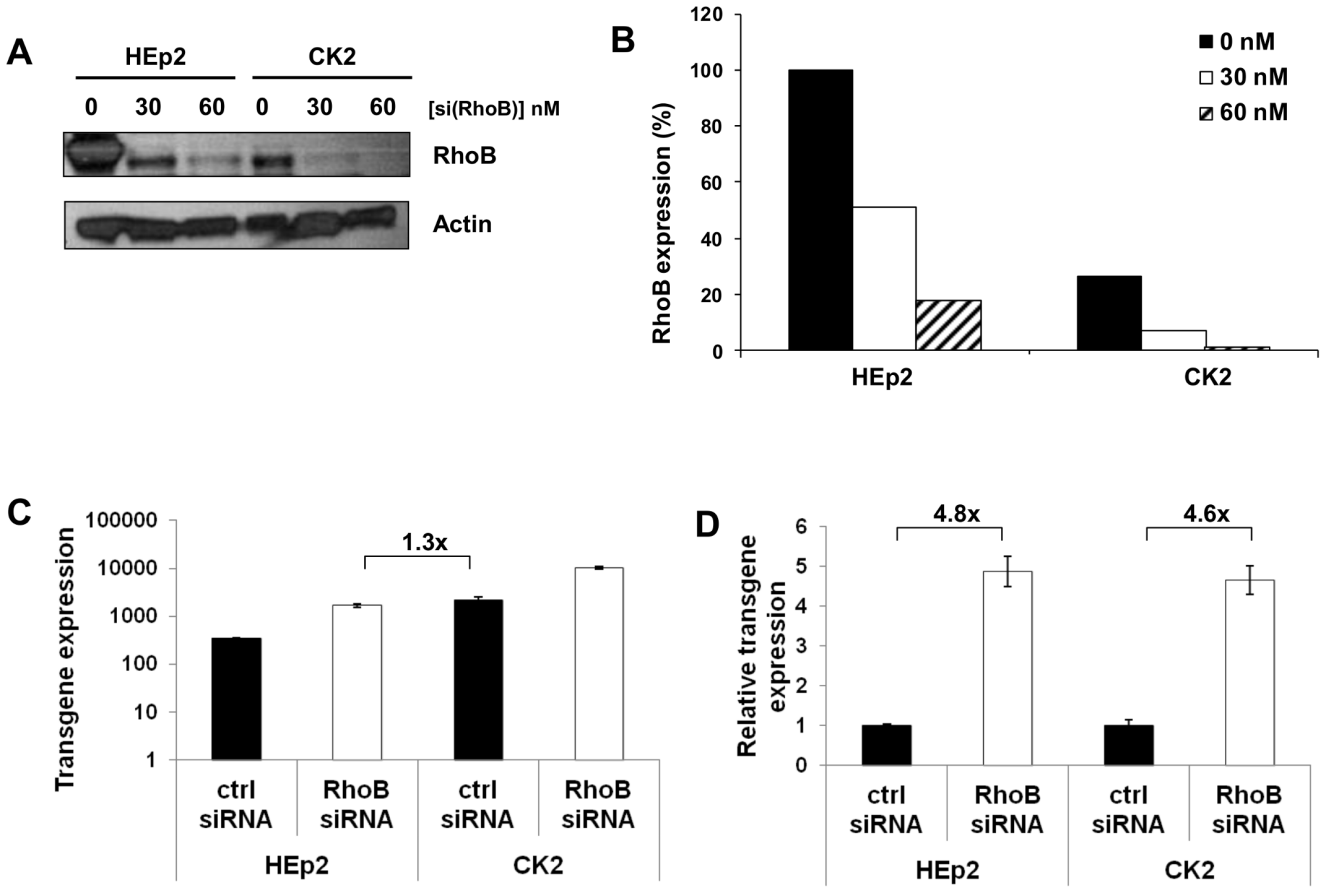
Decreasing RhoB expression increases Ad5wt-mediated transgene expression. HEp2 and CK2 cells were transfected with control or RhoB siRNA. Twenty-four hours after transfection cells were replated in 96-well plates for Ad5wt-mediated transgene expression measurement and 6-well plates for Western blot. Forty-eight hours after siRNA transfection, the level of RhoB was analyzed by Western blot and cells in 96-well plates were transduced for 1 hour at 37°C with two-fold serial dilutions of Ad5wt. Twenty-four hours after infection, cells were stained for β-galactosidase expression. The transgene expression obtained with an MOI of 10^4^ pp/cell is presented. (A) Forty-eight hours post-transfection the level of RhoB protein was analyzed by Western blot, i.e. at the time of Ad5wt transduction whose results are presented in C and D. Actin was used as a loading control. A representative blot of two independent experiments is presented. (B) Densitometric analysis of Western blot presented in (A). Data are presented as relative to RhoB expression in HEp2 cells that was set as 100%. (C) Ad5wt-mediated transgene expression in HEp2 and CK2 cells transfected with control or RhoB-specific siRNAs. (D). Data shown in (C) are presented as relative to control siRNA in HEp2 and CK2 cells, respectively. The results presented in (C) and (D) are representative of three independent experiments with similar results ± standard deviation.

From the data previously published in our laboratory, we know that RhoB expression is strongly down-regulated in cisplatin-resistant CK2 cells in comparison with HEp2 cells [31]. Bearing in mind the data from the literature showing that RhoB coordinates endosome motility within the cell [18], [36], we wished to investigate whether RhoB expression could influence Ad5wt-mediated transgene expression. We transfected HEp2 and CK2 cells with RhoB-specific siRNA (30 nM and 60 nM) and 48 hours post-transfection confirmed efficacy of RhoB silencing by Western blot (Figure 4 A, B). Since transfection with 60 nM RhoB-specific siRNA completely abolished RhoB expression in CK2 cells, we decided to use 30 nM siRNA to assess the effect of decreased RhoB in an Ad5wt transduction assay. Forty-eight hours after transfection with control or RhoB-specific siRNA, HEp2 and CK2 cells were transduced with Ad5wt. Decreasing RhoB expression in the HEp2 cell line brought Ad5wt-mediated transgene expression very close to the level observed in CK2 cells (Figure 4C). Concomitantly, the level of RhoB in HEp2 cells transfected with RhoB-specific siRNA was close to the level of RhoB in CK2 cells transfected with control siRNA (Figure 4C). Down-regulation of RhoB expression increased Ad5wt-mediated transgene expression in both HEp2 and CK2 cell line, (4.8 and 4.6 -fold, respectively) (Figure 4D).

To test whether the influence of RhoB silencing on transduction by Ad5-based vectors might be a general rule, we performed the same experiment in a human breast carcinoma cell line, MDA-MB-435S. We obtained similar results; that is, decreasing RhoB expression in MDA-MB-435S cell line led to a significant increase in Ad5wt transduction efficacy (Figure S1).

### Decreasing RhoB Expression has No Influence on the Level of CAR and Integrin Expression nor on the Efficiency of Ad5wt Attachment and Internalization

In the literature, there are a few reports connecting RhoB expression with the expression level of integrins. It has been shown that diminishing RhoB expression in different cell models causes reduction in the amount of integrins available on the cell surface, namely β1 in prostate cancer cell line [37] and β2 and β3 in macrophages derived from RhoB−/− mice [38]. These data led us to examine whether silencing RhoB expression in HEp2 and CK2 cells may have increased Ad5-mediated transduction by influencing cell surface expression of Ad5 receptors. We analyzed expression of CAR and integrins involved in adenovirus attachment and internalization in HEp2 and CK2 cells 48 hours after transfection with RhoB-specific and control siRNA. Silencing of RhoB in HEp2 cells did not change expression of any of the analyzed molecules in comparison with non-transfected cells, nor cause additional change in expression of these receptors in the CK2 cell line (Figure S2). Hence, increased transduction in both HEp2 and CK2 cell line after RhoB silencing is unlikely to be due to the modulation of CAR or integrin expression on the cell surface.

**Figure 5:**
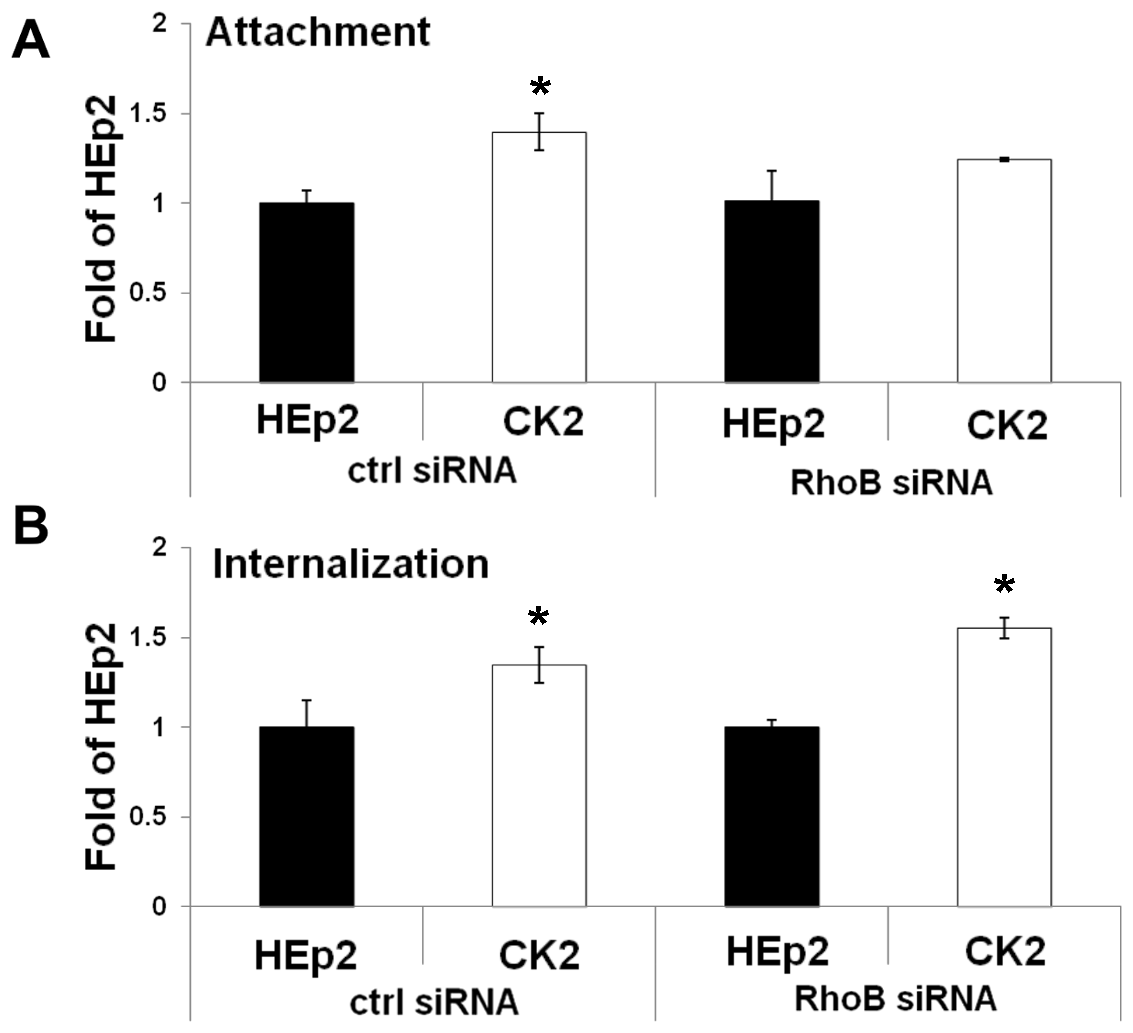
Decreasing RhoB expression by siRNA transfection does not change Ad5wt attachment or internalization. Forty-eight hours after transfection with control or RhoB-specific siRNAs, cells were incubated with Ad5wt at an MOI 1000 pp/cell for 40 minutes on ice. To measure attachment, unbound virus was removed and cells were scraped off and pelleted. To measure internalization, unbound viruses were removed and cells were incubated at 37°C for 40 minutes, trypsinized and pelleted. Total DNA (cellular plus viral) was extracted from cells and used as a sample for quantification of viral DNA by real-time PCR using a region within hexon as the target sequence. The cell-derived DNA content per PCR reaction was normalized by a second real-time PCR assay targeting the GAPDH gene. (A). Attachment of Ad5wt in HEp2 and CK2 cells transfected with control or RhoB-specific siRNAs presented as fold of HEp2. (B). Internalization of Ad5wt in HEp2 and CK2 cells transfected with control or RhoB-specific siRNAs presented as fold of HEp2. The results presented are representative of three independent experiments with similar results ± standard deviation. Asterisks indicate significant differences (*, P<0.05; **, P<0.01).

While RhoB silencing did not change the expression of major Ad5 receptors, we wished to test whether RhoB silencing might nevertheless affect Ad5 attachment and internalization. After transfected with RhoB-specific or control siRNA, we observed that the difference in Ad5wt attachment between CK2 and HEp2 cells was 1.3- or 1.4-fold, respectively (Figure 5A), that is, indistinguishable from the difference in attachment observed in non-transfected CK2 and HEp2 cells (1.5-fold) presented in Figure 3. Thus, transfection itself did not affect the relative efficiency of attachment in these cell lines.

As presented in Figure 3, the difference in Ad5wt internalization between non-transfected CK2 and HEp2 cells was approximately 1.8-fold. Transfection of these cell lines with either RhoB-specific or control siRNA gave rise to a comparable difference in internalization, approximately 1.5-fold for RhoB siRNA and 1.4-fold for control siRNA (Figure 5B). Thus, while transfection itself may have affected Ad5wt internalization and reduced the difference between CK2 and HEp2, the reduction was unrelated to RhoB silencing, as RhoB-specific and control siRNA produced the same extent (a 1.5- and 1.4-fold difference, respectively) (Figure 5B).

We conclude that the role of RhoB in differential transgene expression is unrelated to attachment and internalization. Moreover, Ad5wt attachment and internalization in HEp2 cells were the same after transfection with RhoB-specific or control siRNA. The same observation held for CK2 cells, indicating that silencing RhoB per se affected neither Ad5wt attachment nor internalization, regardless of the cell line. The fact that silencing of RhoB in HEp2 and CK2 cells dramatically increased Ad5wt-mediated transgene expression, which is not the consequence of modulation of expression of cell-surface receptors or change in Ad5wt attachment and/or internalization, implies that RhoB may play a role in post-internalization events, and very possibly in intracellular trafficking.

### Ad5wt Trafficking Differs in CK2 and HEp2 Cell Lines

**Figure 6:**
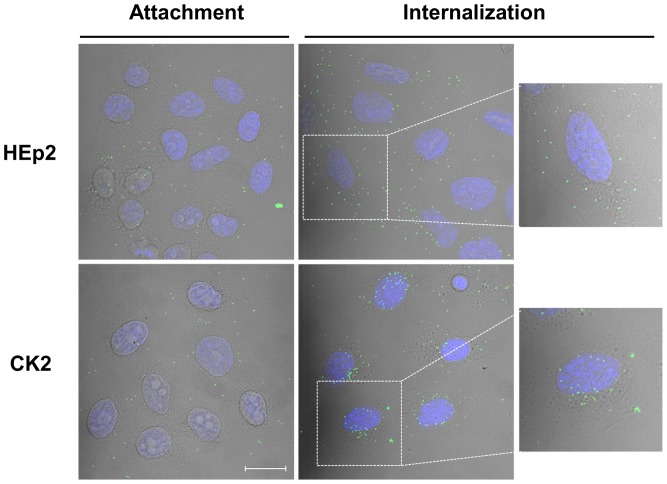
RhoB influences Ad5wt intracellular trafficking. Confocal microscopy of attachment and internalization of fluorescently labeled Ad5wt at MOI 4×10^4^ pp/cell in HEp2 and CK2 cells. Cells were incubated with Alexa Fluor 488-labeled virions for 60 minutes on ice. Unbound virus was removed and cells were either fixed to measure attachment or fed with fresh medium and incubated at 37°C for 30 minutes before fixation to allow virus internalization. Overlay of virus (green), nucleus stained with DRAQ5 (blue) and phase-contrast is shown. Scale bar presents 20 µm.

It is widely accepted that adenovirus cell entry and translocation to the nucleus is a rather quick event. Internalization *via* coated pits occurs within 5 min, efficient liberation from endosomes within 15 min, and 35–45 min after initiation of entry viruses are found at nuclear pore complexes [39]. In order to study intracellular trafficking of Ad5wt in HEp2 and CK2 cell lines, we followed internalization of fluorescently labeled Ad5wt by confocal microscopy. We observed Ad5wt attachment after 40 min incubation on ice, and internalization of attached Ad5wt during 30 minutes after initiation of Ad5wt entry achieved by transfer of cells to 37°C. Labeled Ad5wt particles can be clearly seen attached to the surface of both HEp2 and CK2 cell lines (Figure 6). We were unable to observe significant differences in the number of attached particles, although it seemed that in most of the photographs a slightly higher number of viruses were attached to the CK2 cell surface, in agreement with attachment data obtained by real-time PCR. The most noticeable difference between HEp2 and CK2 cells was visible at 30 minutes after initiation of internalization. As can be seen in Figure 6, while in CK2 cells all viruses were located at the very border of the nucleus at this time point, in HEp2 cells most of the viruses were still scattered in the cytoplasm, with only a few being located close to the nucleus. Adenovirus particles have been shown to be transported toward the microtubule organizing center (MTOC), which is located at a perinuclear position [40]. In the CK2 cell line the position of Ad5wt labeled particles at 30 minutes after initiation of internalization was consistent with localization to the MTOC (Figure 6). The fact that fewer viruses reached the nucleus in HEp2 cells may explain increased Ad5wt-mediated transgene expression in CK2 cells.

All together, our results obtained by measuring Ad5 attachment, internalization and Ad5-mediated transgene expression strongly suggest that RhoB plays a role in Ad5 transport from cell membrane to the nucleus. We have put considerable effort into characterizing trafficking of Ad5wt in HEp2 and CK2 cells after RhoB silencing. Although in most experiments we have seen more viruses located close to the nucleus after RhoB silencing, indicating that RhoB can interfere with successful transport of Ad5 to the nucleus, we have been unable to quantify these changes. Characterization of the role of RhoB in transgene expression of Ad5 will require in-depth study of the intracellular transport of Ad5 after RhoB silencing, which is beyond the scope of the present work.

## Discussion

One of the standard cancer therapies is chemotherapy, for which development of drug resistance to chemotherapy presents a major challenge. In the last decade there have been several studies reporting a beneficial effect from combined adenovirus-mediated gene therapy and chemotherapy in cancer treatment [23], [24], [25], [41]. It has been shown that Ad5-mediated transgene expression is increased in several drug-resistant cell lines selected by exposure to antitumor drugs. For instance, it has been reported that Ad5-mediated gene transfer is more efficient in drug-resistant human bladder cancer cells, making them more sensitive to Ad5-vectored p53 gene therapy than drug-sensitive cells [42]. A few reports in the literature have addressed the molecular mechanisms involved [27, 28, present work]. In our previous study we show that in the cisplatin-resistant HEp2-derived cell clone CA3_ST_, increased Ad5-mediated transgene expression is secondary to up-regulated expression of CAR and αvβ3 integrin [27]. We have also described a cell model in which the level of Ad5-mediated transgene expression was uncoupled from Ad5 attachment/internalization. In particular, in the HEp2-derived vincristine-resistant cell line VK2, increased transgene expression after transduction with Ad5wt is caused by increased activity of the RSV promoter used to drive transgene expression. While the amount of internalized Ad5 DNA was essentially the same in VK2 and parental HEp2 cell lines, Ad5-mediated transgene expression driven by the RSV promoter was significantly increased in VK2 cells (approximately 5-fold) [28]. Here, in a new cell model consisting of parental HEp2 and the cisplatin-resistant cell clone CK2, we have identified RhoB expression as a cause of differential Ad5wt-mediated transgene expression.

Compared with parental HEp2 cells, cisplatin-resistant CK2 cells showed increased Ad5wt-mediated transgene expression accompanied by moderately increased Ad5wt attachment and internalization. While the difference in Ad5wt-mediated transgene expression was 5-fold, the difference in attachment (approximately 1.5-fold) and internalization (approximately 1.5- to 1.8-fold) was much less pronounced, suggesting that these two events could not entirely account for increased Ad5wt-mediated transgene expression in the CK2 cell line. We showed that cisplatin resistance in CK2 cells was accompanied by change in expression of several cell surface molecules involved in adenovirus infection. In CK2 cells, as compared with parental HEp2 cells, we observed increased expression of CAR, the integrin heterodimer α3β1 and integrin subunits β1 and α5, as well as down-regulation of integrin heterodimer αvβ5. We have tested the capacity of CAR up-regulation to enhance Ad5wt-mediated transgene expression in parental HEp2 cells by isolating a cell clone overexpressing CAR. In this instance, a 30% increase in the level of CAR led to an approximately 1.7 fold increase in Ad5wt transduction (data not shown). Increased expression of α5 and β1 subunits in CK2 implies increased expression of α5β1 integrin, which could also contribute to increased attachment and internalization. Therefore, it is likely that increased attachment/internalization of Ad5wt in CK2 cells was the consequence of increased binding to the high affinity receptor CAR and low affinity receptor α5β1. As regards the integrin αvβ5, it can be a limiting factor for transgene expression in HEp2 cells, as we have shown that Ad5wt-mediated transgene expression is diminished when integrin αvβ5 is down-regulated [11]. It is thus unlikely that the diminution in αvβ5 in the CK2 cell line is responsible for increased attachment/internalization in the CK2 cell line. Finally, while the role of α3β1 integrin in adenovirus infection has received little attention, one report indicates that α3β1 integrin is able to interact with penton base in vitro, with the RGD motif being only one of multiple binding sites [6]. Since no change was observed in Ad5-mediated transgene expression in HEp2 and CK2 cells upon transfection with α3-specific siRNA (data not shown), we have concluded that α3β1 integrin is not responsible for increased Ad5-mediated transgene expression in CK2 cells. Additional evidence that differential receptor usage is not responsible for increased Ad5-mediated transgene expression in the CK2 cell line is provided by experiments with capsid-modified Ad5-based vectors (Ad5Δ639, Ad5RGD4C and Ad5Δ639RGD4C). Irrespective of their modification, all vectors gave rise to a comparable increase in Ad5-mediated transgene expression in cisplatin-resistant CK2 cells. The classical adenovirus infection pathway can be roughly divided into five stages that include binding, entry, endosomal escape, translocation, and nuclear import [43]. For optimal adenovirus-mediated transgene expression, all of these steps must be efficient. Since the difference in Ad5-mediated transgene expression between CK2 and HEp2 cells was much higher than the difference in attachment/internalization, we hypothesized that increased Ad5-mediated transgene expression in CK2 cells might have been due to changed intracellular trafficking.

Besides integrins, adenovirus entry requires assembly of clathrin-coated pits and invaginations of the plasma membrane. These processes are all regulated by lipid kinases, actin-modulating small GTPases and the large GTPase dynamin [13], [44]. Recently, it has been shown that down-regulation of RhoB is one of the determinants of cisplatin resistance in CK2 cells [31]. Since CK2 cells durably display morphological differences with HEp2 cells [30], it is possible that they have undergone changes in the cytoskeleton that influence adenovirus internalization. Rho GTPases, members of the Ras superfamily, are small GTP/GDP-binding proteins that are found in all eukaryotes and are involved in regulation of the actin cytoskeleton. It has been shown that Rho GTPases also play an important role in a number of physiological processes, such as membrane trafficking, transcriptional control, regulation of cell adhesion, and cell cycle progression [14]. The best studied Rho GTPases are RhoA, Rac and CdC42. Three highly homologous isoforms, RhoA, RhoB and RhoC, induce stress fibers. Rac1 stimulates lamellipodium and membrane ruffle formation, while Cdc42 participates in regulation of cell polarity and formation of filopodia. While RhoA and RhoC exert their role in the cytosol, RhoB was the first member of the Rho family found to localize to intracellular membrane vesicles [45], and more particularly, endosomes [46]. Through its interaction with endosomes, RhoB delays EGF-induced EGF-R degradation by inhibiting the transfer from late endosomes to lysosomes [47], while oscillation of RhoB GTPase activity is essential for appropriate sorting decisions, and for directing CXCR2 chemokine receptor degradation and recycling – events that are required for optimal chemotaxis [19]. Finally, RhoB has been shown to regulate endosome transport by promoting actin assembly on endosomal membranes [18].

Given the role of RhoB in endosome sorting, we thought that the difference in Ad5wt-mediated transgene expression between HEp2 and CK2 cell lines might be related to differential RhoB expression. Indeed, after transfecting HEp2 and CK2 cells with RhoB siRNA we observed significantly increased Ad5wt-mediated transgene expression in both cell lines. Moreover, increased Ad5wt-mediated transgene expression in HEp2 and CK2 cells was not caused by increased attachment or internalization, indicating that the difference in Ad5-mediated transgene expression between HEp2 and CK2 might be caused by a change in intracellular trafficking of viral particles. Our conclusion that RhoB is involved in Ad5wt-mediated transgene expression was reinforced by the similar results obtained in the MDA-MB-435S cell line.

It is generally accepted that after docking to the CAR receptor Ad5 binds αv integrins and enters the cell by clathrin-mediated endocytosis. After being internalized in endosomal vesicles, adenovirus particles must escape from the endosome. Once situated in the cell cytoplasm, adenovirus encounters an abundance of molecules that impair its free diffusion. Thus, the adenovirus requires some form of active transport to reach the nucleus. Microtubules are cytoskeletal structures responsible for various kinds of movements in all eukaryotic cells. Adenoviruses use microtubules to travel toward the nucleus where they subsequently dock with the nuclear pore complex. After being attached to the microtubules, adenovirus particles travel toward the microtubule organizing center (MTOC), which is typically located at a perinuclear position [48].

In order to gain insight into intracellular trafficking of Ad5wt in HEp2 and CK2 cell lines, we followed the fate of fluorescently labeled Ad5wt by confocal microscopy. Thirty minutes after initiation of internalization in CK2 cells, adenoviruses were observed to be situated in close proximity to the nucleus, in a location characteristic of MTOC. By contrast, in HEp2 cells adenoviruses were found scattered throughout the cytoplasm, suggesting far less efficient nuclear transport. Our observation is in accordance with a study performed by Fernandez-Borja and colleagues [18] in which they have shown that the activated form of endosomal RhoB promotes polymerization of an actin coat around early endosomes and induces their association with subcortical actin fibers, thereby preventing their transport to the juxtanuclear area. Endosomes dispersed in this manner show reduced motility. In the same study, the authors hypothesized that inactivation of RhoB prevents further actin polymerization, and that depolymerization of the actin coat allows endosomes to bind to microtubules *via* the minus-end directed motor dynein for transport toward the microtubule minus-end [18]. Additional support comes from a recently published work from Lajoie-Mazenc and colleagues [49], in which they establish a direct link between RhoB and microtubules. They identified the light chain of the microtubule-associated protein MAP1A (LC2) as a novel binding partner of RhoB, postulating that interactions between RhoB and MAP1A/LC2 facilitate endocytotic vesicle trafficking and regulate the trafficking of signaling molecules.

To the best of our knowledge, this is the first study that shows an important role for RhoB in Ad5-mediated transgene expression. We show here that down-regulation of RhoB significantly increased Ad5-mediated transgene expression in HEp2 and CK2 cells, without changing the amount of attached or internalized Ad5wt in either cell line. We showed that down-regulation of RhoB in HEp2 cells to a level similar to that in CK2 cells increased Ad5-mediated transgene expression in HEp2 to the level observed in CK2. We also observed different Ad5wt trafficking pattern between HEp2 and CK2 cells. Based on our results we conclude that increased Ad5-mediated transgene expression in CK2 cells as compared with HEp2 cells is due also to altered Ad5wt trafficking from membrane to nucleus. We hypothesize that this is caused by diminished RhoB expression. The detailed mechanisms underlying this phenomenon remain to be determined.

Our results may have important clinical implications in that they provide further evidence suggesting that development of drug resistance, while representing a major obstacle in standard cancer treatment, may cause changes that can be rather beneficial for alternative therapeutic approaches, such as gene therapy. Here we show that decreased RhoB expression, which was identified as a cause of cisplatin resistance in the CK2 cell line, is also responsible for increased Ad5-mediated transgene expression that is unrelated to increased entry of Ad5. This feature of drug-resistant cells might be gainfully exploited in cancer gene therapy of drug-resistant tumors with down-regulated RhoB expression. In particular, enhanced Ad5-mediated transgene expression, secondary to diminished expression of RhoB, may enable reduction in the required dose of Ad5 in clinical applications.

## Supporting Information

Figure S1 **Decreasing RhoB expression increases Ad5-mediated transgene expression in human breast carcinoma MDA-MB-435S cell line.** Forty-eight hours after transfection with siRNA, cells were plated in 96-well plates and, 24 hours later, transduced for 1 hour at 37°C with two-fold serial dilutions of Ad5wt. Twenty-four hours after transduction, cells were stained for β-galactosidase expression. The transgene expression obtained with an MOI of 10^4^ pp/cell is presented. The data presented are representative of three independent experiments with similar results ± standard deviation.
(TIF)

Figure S2 **Cell surface levels of CAR, integrin heterodimers α3β1, integrin subunits α5 and β1 on HEp2 and CK2 cells after transfection with control or RhoB specific siRNA.** Cells were detached by Versene and incubated with murine monoclonal antibodies or isotype-matched antibody as a negative control. After incubation with the secondary reagent (PE-conjugated anti-mouse antibody), labeled cells were analyzed by flow cytometry. Mean fluorescence intensities relative to untransfected HEp2 cells obtained in three independent experiments that gave similar results are shown.
(TIF)
