# Supplementary figures and images for: Increased Adenovirus Type 5 Mediated Transgene Expression Due to RhoB Down-Regulation
Source: PLoS One. 2014 Jan 22;9(1):e86698. doi: 10.1371/journal.pone.0086698 (PMC3899303; doi:10.1371/journal.pone.0086698)

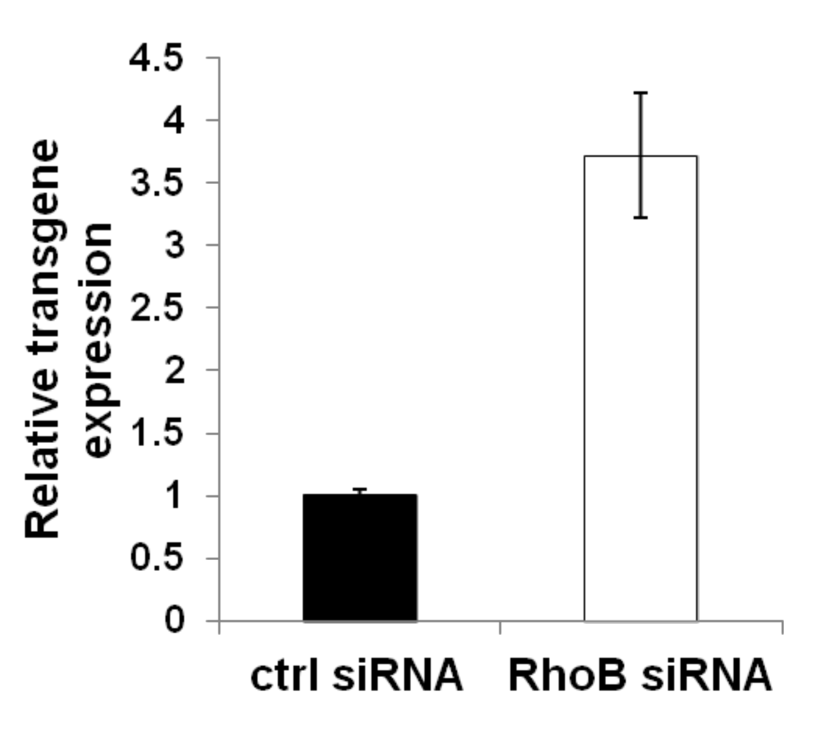

Supplement: Figure S1 — Decreasing RhoB expression increases Ad5-mediated transgene expression in human breast carcinoma MDA-MB-435S cell line. Forty-eight hours after transfection with siRNA, cells were plated in 96-well plates and, 24 hours later, transduced for 1 hour at 37°C with two-fold serial dilutions of Ad5wt. Twenty-four hours after transduction, cells were stained for β-galactosidase expression. The transgene expression obtained with an MOI of 104 pp/cell is presented. The data presented are representative of three independent experiments with similar results ± standard deviation. (TIF) [file pone.0086698.s001.tif]

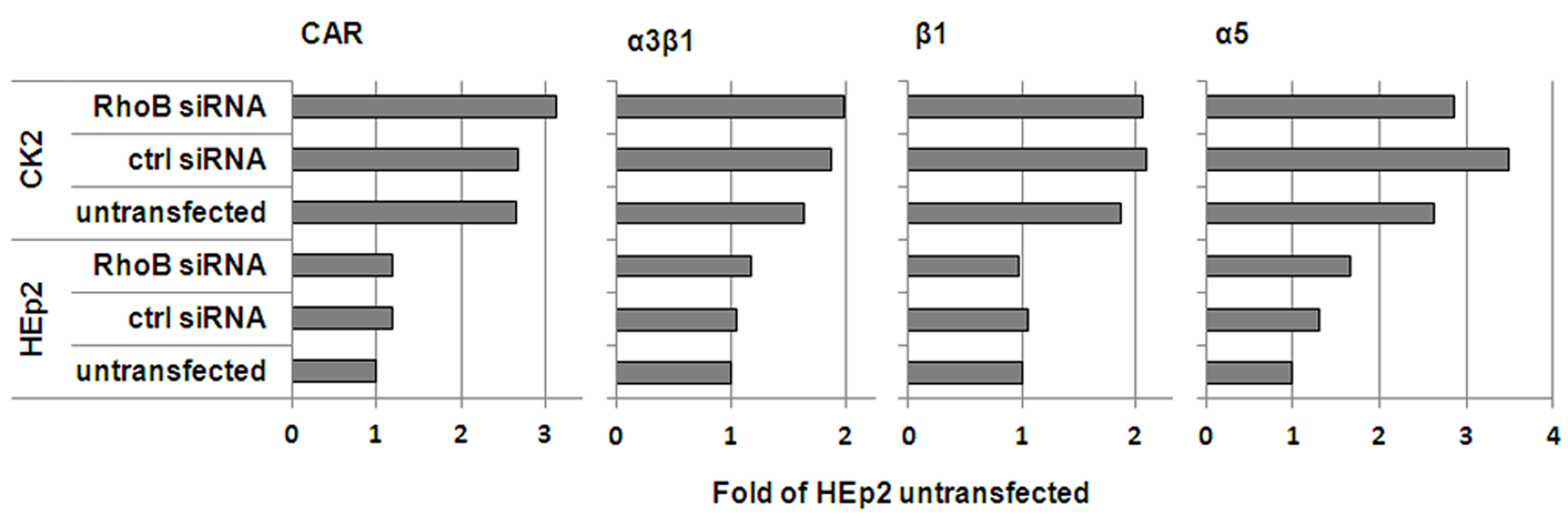

Supplement: Figure S2 — Cell surface levels of CAR, integrin heterodimers α3β1, integrin subunits α5 and β1 on HEp2 and CK2 cells after transfection with control or RhoB specific siRNA. Cells were detached by Versene and incubated with murine monoclonal antibodies or isotype-matched antibody as a negative control. After incubation with the secondary reagent (PE-conjugated anti-mouse antibody), labeled cells were analyzed by flow cytometry. Mean fluorescence intensities relative to untransfected HEp2 cells obtained in three independent experiments that gave similar results are shown. (TIF) [file pone.0086698.s002.tif]
